# Assessment of Susceptibility of Methicillin-Resistant Staphylococcus aureus Isolates to Ceftaroline and Other Antistaphylococcal Agents

**DOI:** 10.7759/cureus.112784

**Published:** 2026-07-16

**Authors:** Rishabh Gupta, Saumya Agarwal, Haniya Jafar, Ved Prakash

**Affiliations:** 1 Department of Microbiology, Rohilkhand Medical College and Hospital, Bareilly, IND

**Keywords:** antimicrobial susceptibility, antistaphylococcal agents, ceftaroline, linezolid, methicillin-resistant, methicillin resistant staphylococcus aureus (mrsa), mrsa, staphylococcus aureus

## Abstract

Introduction

Methicillin-resistant *Staphylococcus aureus* (MRSA) remains a major cause of healthcare-associated infections and is associated with limited treatment options owing to its widespread antimicrobial resistance. This study aimed to evaluate the in vitro susceptibility of clinical MRSA isolates to ceftaroline and determine susceptibility patterns across demographic and clinical variables.

Methods

This cross-sectional observational study included 250 non-duplicate clinical MRSA isolates obtained from various clinical specimens. Antimicrobial susceptibility testing was performed in accordance with the guidelines of the Clinical and Laboratory Standards Institute (CLSI) guidelines. Demographic and microbiological data were analyzed using descriptive statistics, Pearson’s chi-square test or Fisher's exact test, and Cramer's V effect-size measures. Pairwise comparisons between ceftaroline and each comparator antibiotic were conducted using McNemar's test.

Results

Among the 250 MRSA isolates, 158 (63.2%) were recovered from male patients and 92 (36.8%) from female patients. Overall, 212 (84.8%) isolates were susceptible to ceftaroline, 36 (14.4%) demonstrated intermediate susceptibility, and two (0.8%) were resistant. Ceftaroline susceptibility remained consistent across sexes (p=0.847), ages (p=0.312), hospital departments (p=0.862), and specimen types (p=0.931). Vancomycin showed 100% susceptibility, whereas linezolid showed 98.8% susceptibility. Complete resistance to several β-lactam antibiotics was observed.

Conclusion

Ceftaroline demonstrated substantial in vitro activity against clinical MRSA isolates, and remained effective across diverse demographic and clinical subgroups. These findings support its potential role as an additional therapeutic option for MRSA infections and highlight the importance of continued antimicrobial surveillance and susceptibility-guided therapy to monitor the emerging resistance patterns.

## Introduction

Methicillin-resistant *Staphylococcus aureus *(MRSA) remains a formidable pathogen in both community and healthcare settings, posing significant therapeutic challenges worldwide [1]. Since the emergence of MRSA in the 1960s, it has evolved into a major cause of skin and soft tissue infections, bacteremia, pneumonia, and surgical site infections, contributing to increased morbidity, mortality, and healthcare costs [2]. The widespread resistance of MRSA to multiple classes of antibiotics, particularly β-lactams, necessitates the development and evaluation of new antimicrobial agents with enhanced activities against these resistant strains [3].

Ceftaroline, a fifth-generation cephalosporin, is a promising therapeutic option because of its unique ability to bind penicillin-binding protein 2a (PBP2a), which confers methicillin resistance to *S. aureus* [4,5]. Unlike earlier β-lactams, ceftaroline demonstrated potent in vitro activity against MRSA, while retaining activity against methicillin-susceptible strains. Linezolid, an oxazolidinone antibiotic, is a well-established comparator with excellent efficacy against MRSA, particularly in complicated skin and soft tissue infections, and pneumonia. However, concerns regarding emerging resistance, toxicity profiles, and cost-effectiveness highlight the need for the ongoing surveillance of newer agents such as ceftaroline in diverse clinical populations [6].

This study was conducted at a tertiary care hospital to evaluate the in vitro efficacy of ceftaroline against clinical MRSA isolates. It aimed to compare the efficacy of ceftaroline with linezolid and other commonly used antibiotics against *S. aureus* and MRSA isolates. The main objectives of this study were to assess the susceptibility pattern of MRSA isolates to ceftaroline, evaluate the distribution of ceftaroline susceptibility across various demographic and clinical variables, such as age, sex, hospital departments, and specimen sources, and compare the in vitro activity of linezolid and other antistaphylococcal agents. Additionally, it also aimed to identify statistically significant associations between ceftaroline susceptibility and different patient-related factors.

## Materials and methods

Study design and setting

This study was conducted in the Department of Microbiology, Rohilkhand Medical College and Hospital, Bareilly, Uttar Pradesh, India. It was conducted over a period of one year between August 2024 and August 2025. Clinical specimens obtained from inpatient wards, intensive care units, emergency services, and outpatient departments were processed in a bacteriology laboratory according to standard microbiological procedures.

Ethical considerations

The study protocol was reviewed and approved by the Institutional Ethics Committee (IEC), Rohilkhand Medical College and Hospital, Bareilly, Uttar Pradesh, India (approval no. IEC/RMCH/002/2024/JUL; approval date: July 20, 2024). It involved the microbiological analysis of clinical isolates obtained as a part of routine patient care. Patient confidentiality was maintained throughout the study and all data were anonymized prior to analysis.

Study population

Clinical specimens submitted to the Department of Microbiology for routine culture and antimicrobial susceptibility testing during the study period were screened for *S. aureus*. MRSA isolates identified from these specimens were included in this study.

Eligibility criteria

Clinical specimens received in the Department of Microbiology from both outpatients and hospitalized patients during the study period were screened for *S. aureus*. Non-duplicate MRSA isolates recovered from various clinical specimens, including blood, pus, urine, sputum, bronchoalveolar lavage fluid, endotracheal aspirates, cerebrospinal fluid, pleural fluid, and other body fluids, were considered eligible for inclusion in the study. Duplicate isolates obtained from the same patient and specimen source, as well as isolates identified as microorganisms other than MRSA were excluded from the analysis.

Sample size estimation

The sample size was estimated using the G*Power software (version 3.1, Heinrich-Heine-Universität Düsseldorf, Düsseldorf, Germany). For a one-group proportion analysis, based on an expected ceftaroline susceptibility rate of 95% among MRSA isolates (derived from preliminary data), with a 95% confidence level (CI) and 3% absolute margin of error, the minimum required sample size was calculated as 205 isolates [7]. To account for an anticipated 10% loss due to incomplete data or invalid results, the final sample size was adjusted to 250 MRSA isolates.

Isolation and identification of *S. aureus*

Clinical specimens were processed according to standard microbiological protocols. Direct Gram staining was performed as previously described. The specimens were cultured on blood, MacConkey, and nutrient agar plates and incubated aerobically at 37^0^C for 18-24 h. The presumptive identification of *S. aureus* was based on colony morphology, Gram staining, and conventional biochemical tests. Colonies demonstrating gram-positive cocci arranged in clusters, catalase positivity, coagulase positivity, and characteristic golden-yellow pigmented growth with β-hemolysis on blood agar were identified as *S. aureus*.

Detection of MRSA

All confirmed *S. aureus* isolates were screened for methicillin resistance using the cefoxitin (30 µg) disk diffusion test on Mueller-Hinton agar according to the Clinical and Laboratory Standards Institute (CLSI) guidelines. A 0.5 McFarland bacterial suspension was inoculated onto Mueller-Hinton agar, and cefoxitin (30 µg) disks were applied. Plates were incubated aerobically at 35 ± 2^o^C for 16-18 hours. Isolates showing a cefoxitin inhibition zone diameter of ≤21 mm were classified as MRSA, whereas those with a zone diameter of ≥22 mm were considered methicillin-susceptible *S. aureus* (MSSA). *S. aureus* ATCC 25923 and MRSA ATCC 43300 were used as quality control strains.

Antimicrobial susceptibility testing

Antimicrobial susceptibility testing was performed using the Kirby-Bauer disk diffusion method on Mueller-Hinton Agar, following CLSI guidelines [8,9]. The evaluated antimicrobial agents were penicillin, ampicillin, amoxicillin, ampicillin-sulbactam, piperacillin-tazobactam, amoxicillin-clavulanate, ceftazidime, cefepime, cefotaxime, ceftriaxone, cefuroxime, gentamicin, ciprofloxacin, levofloxacin, erythromycin, clindamycin, tetracycline, doxycycline, cotrimoxazole, minocycline, linezolid, vancomycin, nitrofurantoin, and ceftaroline.

Determination of ceftaroline susceptibility

The in vitro activity of ceftaroline against MRSA isolates was evaluated using standardized susceptibility testing procedures. The results were interpreted according to CLSI breakpoint criteria and categorized as susceptible, intermediate, or resistant. The primary outcome measure was the proportion of MRSA isolates susceptible to ceftaroline.

Data collection

Demographic and clinical information, including age, sex, hospital department, and specimen source, were recorded using a structured data collection protocol. Antimicrobial susceptibility results for each MRSA isolate were documented and entered into a database for analysis.

Statistical analysis

Demographic and clinical data, including patient age, sex, hospital department, and specimen source, were recorded using structured proforma. Statistical analysis was performed using IBM SPSS Statistics for Windows, Version 25 (Released 2017; IBM Corp., Armonk, New York, United States). Descriptive statistics were used to summarize the categorical variables as frequencies and percentages. Chi square test or Fisher's exact test was applied to evaluate the associations between ceftaroline susceptibility patterns and variables such as age group, sex, hospital department, and sample source. Susceptibility results were dichotomized as susceptible (S) and non-susceptible (NS, comprising intermediate and resistant isolates). Since each MRSA isolate was tested against all antimicrobial agents, paired categorical analyses were performed. Overall differences in susceptibility among antibiotics were assessed using Cochran's Q test. Pairwise comparisons between ceftaroline and each comparator antibiotic were conducted using McNemar's test with Bonferroni-adjusted p-values. Statistical significance was set at p<0.05.

## Results

In total, 250 non-duplicate clinical MRSA isolates were included in this study. Of these, 158 (63.2%) were recovered from male patients, and 92 (36.8%) were recovered from female patients. The mean age of male patients was 46.58 ± 21.07 years, whereas the mean age of female patients was 41.21 ± 18.90 years (Table 1).

**Table 1:** Demographic characteristics of patients with methicillin-resistant Staphylococcus aureus isolates Data are presented as frequency (percentage) or mean ± standard deviation (SD).

Parameter	Sex	Frequency (%)	Mean ± SD
Age (years)	Male	158 (63.2)	46.58 ± 21.07
	Female	92 (36.8)	41.21 ± 18.90

**Figure 1:**
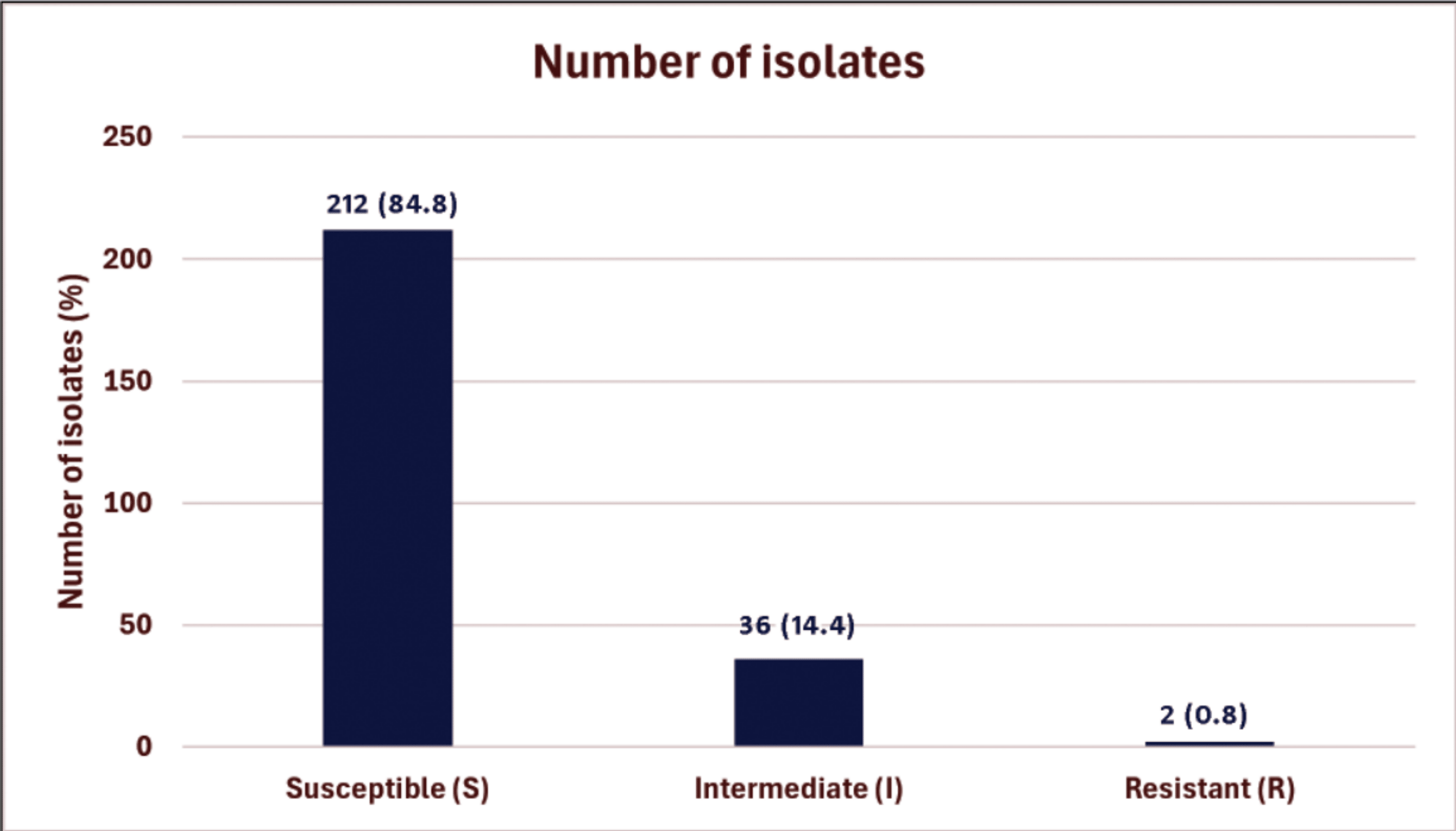
Susceptibility analysis of the MRSA isolates MRSA: Methicillin-resistant Staphylococcus aureus.

The overall susceptibility analysis of the 250 MRSA isolates demonstrated a high level of activity of ceftaroline against the tested organisms. A total of 212 (84.8%) isolates were classified as susceptible, while 36 (14.4%) exhibited intermediate susceptibility. Only two (0.8%) isolates were found to be resistant to ceftaroline (Figure 1).

Regarding sex, 135 (85.4%) isolates obtained from male patients and 77 (83.7%) isolates obtained from female patients were susceptible to ceftaroline. Intermediate susceptibility was observed in 22 (13.9%) male isolates and 14 (15.2%) female isolates, whereas resistance was detected in one (0.6%) isolate from male and one (1.1%) from a female patient (Table 2).

**Table 2:** Association between patient sex and ceftaroline susceptibility among methicillin-resistant Staphylococcus aureus isolates Data are presented as frequency (percentage), p value calculated with Fisher–Freeman–Halton exact (preferred because several expected counts are <5), effect size was estimated using Cramer's V, df: degree of freedom, statistical significance was set at p<0.05.

Gender	Susceptible; n (%)	Intermediate; n (%)	Resistant; n (%)	Total; n (%)	p-value	Effect size
Male	135 (85.4)	22 (13.9)	1 (0.6)	158 (63.2)	0.847	0.03
Female	77 (83.7)	14 (15.2)	1 (1.1)	92 (36.8)		

No statistically significant association was observed between sex and ceftaroline susceptibility (p=0.847), with negligible effect size (Cramer's V=0.03).

Age-group analysis showed that the highest number of isolates originated from patients aged 41-60 years, followed by those aged 21-40 years and 61-80 years. Susceptibility to ceftaroline remained consistently high across all age categories. No significant association was identified between age and ceftaroline susceptibility pattern (p=0.312; Cramer's V=0.13; Table 3).

**Table 3:** Association between age group and ceftaroline susceptibility among methicillin-resistant Staphylococcus aureus isolates Data are presented as frequency (percentage), p-value calculated with Fisher–Freeman–Halton exact (preferred because several expected counts are <5), effect size was estimated using Cramer's V, df: degree of freedom, statistical significance was set at p<0.05.

Age group (years)	Susceptible; n (%)	Intermediate; n (%)	Resistant; n (%)	Total; n (%)	p-value	Effect size
0–20	32 (12.8)	3 (1.2)	0 (0.0)	35 (14.0)	0.312	0.13
21–40	57 (22.8)	10 (4.0)	0 (0.0)	67 (26.8)		
41–60	73 (29.2)	11 (4.4)	0 (0.0)	84 (33.6)		
61–80	47 (18.8)	12 (4.8)	2 (0.8)	61 (24.4)		
>80	3 (1.2)	0 (0.0)	0 (0.0)	3 (1.2)		
Total	212 (84.8)	36 (14.4)	2 (0.8)	250 (100)		

Analysis according to the hospital department revealed that the highest number of MRSA isolates were recovered from the Casualty Department, followed by the Department of Medicine. Ceftaroline susceptibility remained high across all departments, and no statistically significant association was found between the hospital department and susceptibility patterns (p=0.862; Cramer's V=0.20) (Table 4).

**Table 4:** Association between the hospital department and ceftaroline susceptibility among methicillin-resistant Staphylococcus aureus isolates Data are presented as frequency (percentage), p-value calculated with Fisher–Freeman–Halton exact (preferred because several expected counts are <5), effect size was estimated using Cramer's V, df: degree of freedom, statistical significance was set at p<0.05.

Department	Susceptible; n (%)	Intermediate; n (%)	Resistant; n (%)	Total; n (%)	p-value	Effect size
Ortho	18 (7.2)	1 (0.4)	0 (0.0)	19 (7.6)	0.862	0.20
Surgery	23 (9.2)	4 (1.6)	0 (0.0)	27 (10.8)		
Medicine	56 (22.4)	8 (3.2)	1 (0.4)	65 (26.0)		
Casualty	68 (27.2)	17 (6.8)	1 (0.4)	86 (34.4)		
Radiation	7 (2.8)	2 (0.8)	0 (0.0)	9 (3.6)		
Pediatrics	14 (5.6)	1 (0.4)	0 (0.0)	15 (6.0)		
ENT	12 (4.8)	0 (0.0)	0 (0.0)	12 (4.8)		
Oncology	0 (0.0)	1 (0.4)	0 (0.0)	1 (0.4)		
Obstetrics & Gynaecology	7 (2.8)	2 (0.8)	0 (0.0)	9 (3.6)		
Chest & TB	4 (1.6)	0 (0.0)	0 (0.0)	4 (1.6)		
Skin	2 (0.8)	0 (0.0)	0 (0.0)	2 (0.8)		
Dental	1 (0.4)	0 (0.0)	0 (0.0)	1 (0.4)		
Total	212 (84.8)	36 (14.4)	2 (0.8)	250 (100)		

When stratified according to specimen source, blood samples accounted for the largest proportion of MRSA isolates, followed by pus samples. High rates of ceftaroline susceptibility were observed across all specimen types. No statistically significant association was identified between specimen source and ceftaroline susceptibility pattern (p=0.931; Cramer's V=0.18; Table 5).

**Table 5:** Association between specimen source and ceftaroline susceptibility among methicillin-resistant Staphylococcus aureus isolates. Data are presented as frequency (percentage), p-value calculated with Fisher–Freeman–Halton exact (preferred because several expected counts are <5), effect size was estimated using Cramer's V, df: degree of freedom, statistical significance was set at p<0.05.

Sample type	Susceptible; n (%)	Intermediate; n (%)	Resistant; n (%)	Total; n (%)	p-value	Effect size
Blood	138 (55.2)	26 (10.4)	1 (0.4)	165 (66.0)	0.931	0.18
Pus	52 (20.8)	5 (2.0)	1 (0.4)	58 (23.2)		
Sputum	1 (0.4)	1 (0.4)	0 (0.0)	2 (0.8)		
Ascitic fluid	1 (0.4)	0 (0.0)	0 (0.0)	1 (0.4)		
Urine	13 (5.2)	3 (1.2)	0 (0.0)	16 (6.4)		
Pleural fluid	3 (1.2)	0 (0.0)	0 (0.0)	3 (1.2)		
Bronchoalveolar lavage fluid	1 (0.4)	0 (0.0)	0 (0.0)	1 (0.4)		
Vaginal swab	1 (0.4)	0 (0.0)	0 (0.0)	1 (0.4)		
Cervical swab	0 (0.0)	1 (0.4)	0 (0.0)	1 (0.4)		
Tissue	1 (0.4)	0 (0.0)	0 (0.0)	1 (0.4)		
Pus (breast)	1 (0.4)	0 (0.0)	0 (0.0)	1 (0.4)		
Total	212 (84.8)	36 (14.4)	2 (0.8)	250 (100)		

The antimicrobial susceptibility profiles of the 250 MRSA isolates to other tested antibiotics are summarized in Table 6.

**Table 6:** Antimicrobial susceptibility profile of the methicillin-resistant Staphylococcus aureus isolates Data are presented as frequency (percentage), susceptibility results were interpreted according to Clinical and Laboratory Standards Institute (CLSI) criteria. Percentages for nitrofurantoin are calculated using only the 16 urinary isolates tested (n=16).

Antibiotic (N = 250)	Susceptible; n (%)	Intermediate; n (%)	Resistant; n (%)
Penicillin (10µg)	0 (0.0)	0 (0.0)	250 (100.0)
Ampicillin (10µg)	0 (0.0)	0 (0.0)	250 (100.0)
Amoxicillin (10µg)	0 (0.0)	0 (0.0)	250 (100.0)
Ampicillin-sulbactam (10/10µg)	0 (0.0)	0 (0.0)	250 (100.0)
Piperacillin-tazobactam (100/10µg)	0 (0.0)	0 (0.0)	250 (100.0)
Amoxicillin- clavulanate (20/10µg)	0 (0.0)	0 (0.0)	250 (100.0)
Ceftazidime(30µg)	0 (0.0)	0 (0.0)	250 (100.0)
Cefepime (30µg)	0 (0.0)	0 (0.0)	250 (100.0)
Cefotaxime (30µg)	0 (0.0)	0 (0.0)	250 (100.0)
Ceftriaxone (30µg)	0 (0.0)	0 (0.0)	250 (100.0)
Cefuroxime (30µg)	0 (0.0)	0 (0.0)	250 (100.0)
Gentamycin (10µg)	133 (53.2)	7 (2.8)	110 (44.0)
Doxycycline (30µg)	172 (68.8)	1 (0.4)	77 (30.8)
Minocycline (30µg)	215 (86.0)	0 (0.0)	35 (14.0)
Tetracycline (30µg)	12 (4.8)	2 (0.8)	236 (94.4)
Ciprofloxacin (5µg)	20 (8.0)	6 (2.4)	224 (89.6)
Levofloxacin (5µg)	26 (10.4)	0 (0.0)	224 (89.6)
Erythromycin (15µg)	23 (9.2)	6 (2.4)	221 (88.4)
Clindamycin (2µg)	95 (38.0)	1 (0.4)	154 (61.6)
Cotrimoxazole (25µg)	97 (38.8)	2 (0.8)	151 (60.4)
Linezolid (30µg)	247 (98.8)	0 (0.0)	3 (1.2)
Vancomycin 0.5µg/ml	250 (100.0)	0 (0.0)	0 (0.0)
Nitrofurantoin (300µg)	13 (81.3)	1 (6.3)	2 (12.5)

Complete resistance was observed to penicillin, ampicillin, amoxicillin, ampicillin-sulbactam, piperacillin-tazobactam, amoxicillin-clavulanate, and multiple cephalosporins. In contrast, vancomycin demonstrated 100% susceptibility, whereas linezolid exhibited excellent activity against 247 (98.8%) susceptible isolates. High susceptibility rates were also observed for minocycline and nitrofurantoin.

The comparative susceptibility analysis revealed that ceftaroline demonstrated significantly superior activity compared to all beta‑lactams, tetracycline, fluoroquinolones, erythromycin, clindamycin, and cotrimoxazole (p<0.0001). Its susceptibility was comparable to minocycline (p=0.76). However, ceftaroline showed significantly lower susceptibility than vancomycin and linezolid (p<0.0001). These findings highlight ceftaroline's excellent in vitro activity against MRSA, though vancomycin and linezolid remain the most universally susceptible agents (Table 7).

**Table 7:** Comparative susceptibility analysis of ceftaroline with each antibiotic N=250, S: Susceptible, NS: Non-susceptible isolates, p-values were calculated using McNemar test (χ²), *p-values were adjusted for multiple comparisons using the Bonferroni correction. The adjusted significance threshold was α=0.05/22 =0.0023.

Antibiotic	Ceftaroline (S/NS)	Antibiotic (S/NS)	McNemar χ²	p-value
Penicillin (10µg)	212 / 38	0 / 250	210.005	<0.0001*
Ampicillin (10µg)	212 / 38	0 / 250	210.005	<0.0001*
Amoxicillin (10µg)	212 / 38	0 / 250	210.005	<0.0001*
Ampicillin-sulbactam (10/10µg)	212 / 38	0 / 250	210.005	<0.0001*
Piperacillin-tazobactam (100/10µg)	212 / 38	0 / 250	210.005	<0.0001*
Amoxicillin-clavulanate (20/10µg)	212 / 38	0 / 250	210.005	<0.0001*
Ceftazidime (30µg)	212 / 38	0 / 250	210.005	<0.0001*
Cefepime (30µg)	212 / 38	0 / 250	210.005	<0.0001*
Cefotaxime (30µg)	212 / 38	0 / 250	210.005	<0.0001*
Ceftriaxone (30µg)	212 / 38	0 / 250	210.005	<0.0001*
Cefuroxime (30µg)	212 / 38	0 / 250	210.005	<0.0001*
Gentamycin (10µg)	212 / 38	133 / 117	69.931	<0.0001*
Doxycycline (30µg)	212 / 38	172 / 78	28.167	<0.0001*
Minocycline (30µg)	212 / 38	215 / 35	0.093	0.76
Tetracycline (30µg)	212 / 38	12 / 238	194.123	<0.0001*
Ciprofloxacin (5µg)	212 / 38	20 / 230	190.005	<0.0001*
Levofloxacin (5µg)	212 / 38	26 / 224	184.005	<0.0001*
Erythromycin (15µg)	212 / 38	23 / 227	187.005	<0.0001*
Clindamycin (2µg)	212 / 38	95 / 155	105.953	<0.0001*
Cotrimoxazole (25µg)	212 / 38	97 / 153	96.267	<0.0001*
Linezolid (30µg)	212 / 38	247 / 3	33.029	<0.0001*
Vancomycin (0.5µg/ml)	212 / 38	250 / 0	36.026	<0.0001*

Nitrofurantoin was excluded from comparative analysis because it was tested only in urinary isolates, resulting in a different denominator from ceftaroline.

## Discussion

The present study demonstrated the high in vitro susceptibility of clinical MRSA isolates to ceftaroline. These findings align with several global and regional studies highlighting their potent activity against MRSA [10]. The unique affinity of ceftaroline for penicillin-binding protein 2a (PBP2a) enables it to overcome the primary mechanism of methicillin resistance, distinguishing it from earlier generation cephalosporins [4,5]. In this study, ceftaroline retained its excellent efficacy despite its universal resistance to other β-lactams, supporting its role as a valuable therapeutic option.

The observed susceptibility rate of 84.8% was consistent with the large-scale surveillance data. A multinational study involving over 35,000 MRSA isolates reported 89.3% susceptibility, with regional variations higher in the United States and Europe but lower in parts of the Asia-Pacific region [11]. Similarly, studies from India and Egypt have reported susceptibility rates exceeding 90%, while some reports have noted slightly lower rates with higher intermediate categories [12,13]. The low resistance rate in the current study is encouraging and comparable to findings in which true resistance remains rare (often <1-3%).

No significant associations were found between ceftaroline susceptibility and demographic or clinical variables such as sex, age group, hospital department, or specimen source. This uniformity suggests that ceftaroline activity is broadly reliable across the diverse patient populations in the study setting. The predominance of isolates from blood and pus samples, together with the high representation from the Casualty and Medicine departments in this study, mirrors the typical epidemiology of MRSA infections in tertiary care settings. These findings are consistent with a recent eight-year retrospective study conducted in a tertiary care hospital in Saudi Arabia, which reported that 68% of MRSA infections were hospital acquired [14]. Bloodstream and deep-seated infections (commonly originating from pus) were prominent, particularly in patients admitted to high-acuity areas. This distribution highlights the role of invasive procedures, prolonged hospitalization, and exposure to critical care in the spread of MRSA in resource-intensive hospital settings.

The antimicrobial susceptibility profile observed in the present study demonstrated that vancomycin and linezolid remained highly active against MRSA isolates, with susceptibility rates of 100% and 98.8%, respectively. These findings are clinically relevant, because both agents continue to represent important therapeutic options for the management of serious MRSA infections. Recent systematic reviews and meta-analyses have reported comparable effectiveness of linezolid and vancomycin in the treatment of MRSA bacteremia and nosocomial pneumonia, with no consistent differences in mortality or overall treatment success rates, although linezolid may offer advantages in specific clinical settings because of its superior tissue penetration and favorable pharmacokinetic profile [15-17]. The sustained susceptibility observed in this study further supports the continued use of these agents against MRSA. In contrast, complete resistance to several β-lactam antibiotics and high rates of resistance to multiple other antimicrobial classes indicate the persistence of multidrug-resistant MRSA strains in the hospital environment. These findings underscore the importance of routine susceptibility testing, antimicrobial stewardship, and continuous surveillance of resistance trends to optimize therapeutic decision-making and preserve the effectiveness of available anti-MRSA agents.

Limitations and clinical implications

This study has certain limitations. This single-center, in vitro investigation was conducted at a tertiary care teaching hospital in northern India, which may limit the generalizability of the findings to other geographical regions and healthcare settings. Furthermore, the cross-sectional design did not permit the assessment of clinical outcomes, microbiological cure rates, treatment response, or the emergence of resistance during therapy. Molecular characterization of MRSA isolates, including the detection of resistance-associated genes and determination of clonal lineages, was not performed. Despite these limitations, our study had several important clinical implications. The high susceptibility of MRSA isolates to ceftaroline supports its potential role as an alternative therapeutic option for the management of MRSA infections, particularly in patients who are intolerant to, unsuitable for, or have failed conventional therapies such as vancomycin or linezolid. Local susceptibility data generated through studies such as the present investigation provides valuable evidence for empirical antibiotic selection and antimicrobial stewardship initiatives. Continued surveillance is essential to monitor evolving resistance patterns and ensure sustained effectiveness of anti-MRSA therapies.

## Conclusions

The present study demonstrated that ceftaroline exhibited substantial in vitro activity against clinical MRSA isolates, with high susceptibility observed across diverse demographic and clinical subgroups. Although vancomycin and linezolid remain the most active agents, ceftaroline may serve as a valuable therapeutic alternative, particularly in patients who are intolerant to or have failed conventional therapies. The complete resistance observed to multiple β-lactam antibiotics highlights the persistence of multidrug-resistant MRSA strains and reinforces the importance of routine susceptibility testing and continuous resistance surveillance. These findings support the potential role of ceftaroline in antimicrobial stewardship programs and empiric antibiotic selection in tertiary care settings. However, multicenter studies correlating in vitro susceptibility with clinical outcomes and incorporating molecular characterization of MRSA isolates are warranted to further validate these findings and monitor emerging resistance trends.
